# The Factors Influencing Public Satisfaction with Community Services for COVID-19: Evidence from a Highly Educated Community in Beijing

**DOI:** 10.3390/ijerph191811363

**Published:** 2022-09-09

**Authors:** Qihui Xie, Xun Xie, Siwei Guo

**Affiliations:** Department of Public Administration, School of Law and Humanities, China University of Mining and Technology (Beijing), Beijing 100083, China

**Keywords:** highly educated residents, services for COVID-19, community governance, satisfaction influence factors

## Abstract

The satisfaction of highly educated citizens with community services for COVID-19 represents the attitude of the middle class and plays an important role in both the social and political stability of a country. The aim of this paper was to determine which factors influence public satisfaction with COVID-19 services in a highly educated community. Through a literature review and using the American Customer Satisfaction Index (ACSI) model, this paper constructed a public satisfaction model of community services for COVID-19 and proposed relevant research hypotheses. A community with many highly educated residents in Beijing was selected as the case study, where 450 official questionnaires were distributed based on the age ratio of residents, with 372 valid questionnaires being collected from May 2021 to July 2021. The study results obtained by a structural equation model (SEM) show that: (1) public satisfaction is significantly and positively influenced by quality perception (0.305 **), public demand (0.295 **), and service maturity (0.465 ***); (2) public satisfaction has a significantly positive effect on service image (0.346 ***) and public trust (0.232 **), and service image significantly affects public trust (0.140 *); (3) service maturity is positively influenced by public demand (0.460 ***) and quality perception (0.323 *); and (4) public demand is positively influenced by quality perception (0.693 ***) (* *p* < 0.05; ** *p* < 0.01; *** *p* < 0.00). The conclusions of the study can provide suggestions and recommendations to improve the satisfaction of highly educated residents with community healthcare services during the COVID-19 pandemic.

## 1. Introduction

In urban and rural areas, community is the smallest component unit of governance. As the government’s refinement of governance has gradually deepened, urban communities have become an important basic unit in urban governance, a basic place to realize social co-governance, and have played an important role in promoting a country’s ability to improve grassroots governance. At the beginning of 2020, the world experienced a major pandemic, with a vast scope and far-reaching effects. The COVID-19 pandemic has taken the lives of millions of people, caused the world to enter economic recession, and has even touched the world’s political and economic structure. The pandemic has become a significant test of social governance in countries around the world. Social governance in China coped relatively well with the COVID-19 pandemic in which community services for COVID-19 played an important role.

A highly educated community is a community in which most of the residents living in it have a bachelor’s degree or higher [1]. It is meaningful to study public satisfaction with COVID-19 services in highly educated communities to determine whether the middle class, represented by the highly educated citizens, is satisfied with the community services for COVID-19 or not, which is important to the social stability of a country [2]. Although some studies in the literature examined public satisfaction with general types of community services [3], less work has been conducted on highly educated communities as a specific type of community to study. In addition, current research on community services in the COVID-19 pandemic is mainly conducted from the community perspective, considering aspects, such as community service resources [4,5,6], community service content [4,7,8], and community service access channels [9,10], but there is a lack of research on community services for COVID-19 from the public perspective, including topics, such as public satisfaction, public demands, public participation, or public perception. This paper therefore poses the following research question: what factors influence public satisfaction with COVID-19 services in a highly educated community? By addressing this research question, not only will a case focused on a highly educated community be added to the case studies of COVID-19 services but the number of works in the literature on community services for COVID-19 from a public perspective will also be increased.

In order to solve the research question, this paper constructed a public satisfaction model of community services for COVID-19, including the antecedent variables of public satisfaction, such as quality perception, service maturity and public demand, as well as the consequence variables of public satisfaction, such as public trust and service image, based on the literature and the ACSI model. Research hypotheses suggesting that public satisfaction is influenced by quality perception, service maturity and public demand, and that public satisfaction affects service image and public trust, were proposed. Hypotheses exploring the relationships within the antecedent variables and consequence variables were also proposed. As a cosmopolitan city, the COVID-19 services in Beijing’s communities have made an outstanding contribution to the city’s pandemic response. A typical feature of Beijing, as the political and economic center of the country, is that it has a high number of highly educated communities. Questionnaire surveys were finished in a highly educated community in Beijing to verify the research hypotheses. A structural equation model was used to analyze the questionnaire data. According to the results of the data analysis and case study, the objectives of this paper were to explore the factors influencing the satisfaction of highly educated residents with community services for COVID-19 and to promote community healthcare services during a major pandemic.

## 2. Literature Review

### 2.1. Community Services for COVID-19

In the current study, research on community services for COVID-19 is divided into four areas: service resources, service content, access to services, and public participation.

#### 2.1.1. Service Resources

Research on service resources mainly included the analysis of human resources, infrastructure and financial support in relation to COVID-19 services. Cheng Y. et al. studied the construction of human resources in COVID-19 services and pointed out the important role played by human resources, such as community organizations and volunteer organizations, in COVID-19 services [3]. The study of Zhang X. and Yang S. found that the construction of infrastructure, such as mobile field hospitals and pandemic detection sites, and the stockpiling of nucleic acid detection kits and personal protective equipment, also had a significant impact on the overall level of community services for COVID-19 [4]. Community services for COVID-19 also require a certain amount of financial support, which comes from government financial funds as well as social charity funds. During the COVID-19 pandemic, the catering, tourism, entertainment, and transportation industries suffered huge losses and the international financial markets were weak, so the economy was negatively affected in the short term [5], which affected financial support for COVID-19 services from the community. However, during the pandemic, many philanthropists and even the general public donated generously, and the charitable funds raised could also provide some support for COVID-19 services from the community [6].

Most of the existing studies on community service resources during the COVID-19 pandemic were only focused on one kind of service resources, and there are many studies on human resources, but only a few on infrastructure or financial support. In addition, a unified study on all three kinds of service resources is missing. This paper will comprehensively study the service resources in terms of these three aspects.

#### 2.1.2. Service Content

Studies on the service content mostly focused on the universality, scientificity, and effectiveness of the community services for COVID-19. The universality of service content refers to whether the services provided by the community during COVID-19 are diverse and comprehensive. Governments around the world took a series of preventive and control measures to slow the spread of COVID-19 in the early days of the pandemic. However, the measures taken by some countries were only a temporary response to the crisis, with a single type of prevention measures and services. In contrast, a wider variety of COVID-19 services were available in communities in China, including checking and registering community visitors, taking people’s temperature, guiding vehicles, calling for and guiding nucleic acid testing, registering nucleic acid testing information, material handling and distribution, health knowledge promotion, etc. [7]. The scientificity of the service content means that the community services for COVID-19 comply with scientific principles. Cable et al. believe that since the SARS pandemic in 2003, China has gradually established an emergency management system for public health emergencies and has accumulated some scientific experience in community services for COVID-19 [8]. The effectiveness of service content refers to the better effect of community COVID-19 services. Zhang and Yang thought that the actual effectiveness of China’s fight against the COVID-19 pandemic also showed that community services were effective in checking suspected patients, preventing the import of pathogenic bacteria, protecting the medical needs of residents, stabilizing public sentiment, and maintaining national security [4].

The existing research on the community service content during the COVID-19 pandemic mostly used qualitative analysis methods and lacks effective data to evaluate the universality, scientificity, and effectiveness of the service content. This paper will provide a quantitative study of these of community service content for COVID-19.

#### 2.1.3. Access to Services

Scholars have mainly explored the diversity, convenience, and effectiveness of community residents’ access to information about services for COVID-19. Some scholars have studied the access to COVID-19 services through questionnaire surveys, such as the study from Jia and Zhu, which showed that people obtain information about community services for COVID-19 through news briefings (91.50%), the Internet (74.05%), WeChat, QQ, and public websites (66.22%) [9]. Some other scholars have studied the convenience and effectiveness of access channels for COVID-19 services in a community. The study from Chan et al. suggests that the public’s perception of the convenience, accuracy, effectiveness, and autonomy of core services will help to improve public satisfaction with community services for COVID-19 [10].

Although the existing surveys have proved that community residents have different access to information about services for COVID-19, they cannot reflect the public’s perception of the quality of these access channels, which will be studied in this paper.

#### 2.1.4. Public Participation

Public participation in the traditional sense focuses on political participation and refers to the process of power sharing between the public and administrative officials, where participation in decision making and public administration contributes to personal development and freedom [11]. Additionally, public participation in the modern sense should include non-political participation, such as social participation and moral participation [12]. Public participation in governance mainly involves urban governance [13], volunteerism [14], corruption governance [15], and public goods redistribution [16].

Public participation in community services for COVID-19 belongs to the dual category of urban governance and public goods redistribution. Most scholars have focused on the activity and effectiveness of public participation in COVID-19 services and the individualized characteristics of COVID-19 services. Baekkeskov E suggested that increasing public participation in COVID-19 services is beneficial for improving the democracy and efficiency of society [17]. Wu et al. thought that the effectiveness of public participation was a necessary prerequisite for achieving high-quality governance [18]. Schoch-Spana et al. argued that COVID-19 services, such as vaccination for special groups and the medical needs of patients with acute illnesses, need to take full account of individual characteristics, and that it is important to pay attention to the individual differences in the public to promote the widespread vaccination of COVID-19 [19].

Existing research on public participation in community services for COVID-19 has focused on the impact of public participation, but few studies have combined public participation and public quality perception. This article will explore the quality perception of public participation in community services.

### 2.2. Public Satisfaction

#### 2.2.1. Measurement of Public Satisfaction

**Figure 1:**
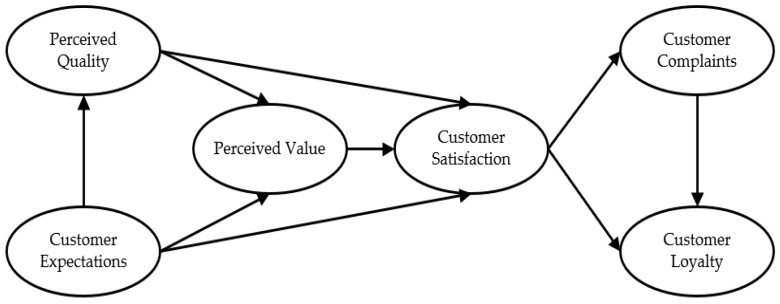
American Customer Satisfaction Index model.

Public satisfaction originated from customer satisfaction theory. The concept of “customer satisfaction” was first introduced in Oliver’s “expectation and paradox” theory. Customer satisfaction is the comparison of the actual results of products and services with the expectations before purchase [20]. Several models of customer satisfaction theory have gradually formed, such as the Sweden Customer Satisfaction Barometer (SCSB), the American Customer Satisfaction Index (ACSI) and the European Customer Satisfaction Index (ECSI), which are in continuous development. Among them, the American Customer Satisfaction Index [21] (as seen in Figure 1) is the most widely used. The model mainly links customer satisfaction and its influencing factors—such as customer expectations, perceived quality and perceived value, etc.—for analysis. Some scholars in public administration have also applied the ACSI model to the study of satisfaction with government public services [22]. This paper will focus on public satisfaction with community services for COVID-19 based on the ACSI model.

#### 2.2.2. Public Satisfaction with Services for COVID-19

Some studies on public satisfaction with services for the COVID-19 pandemic have focused on the antecedent variables influencing satisfaction, such as level of government [23], performance management [24], and sources of performance information [25]. Some other scholars have studied the consequence variables of public satisfaction. Wu C. et. Al., in their study of public satisfaction with the government’s services for COVID-19 in China, suggested that public satisfaction can enhance government image, as well as public trust [26]. Service image and custom trust have already been considered as consequence variables of satisfaction in traditional customer satisfaction studies [27], which has been verified again in studies of COVID-19 services.

At present, the research on public satisfaction with services for COVID-19 has two characteristics: (1) Although some studies have examined the factors influencing public satisfaction with governmental services for COVID-19, fewer have focused on community services. (2) Although some scholars have studied the antecedent variables of public satisfaction and others have studied the consequence variables, few scholars have studied both the antecedent and consequence variables at the same time. This paper will study community services for COVID-19 and will integrate the antecedent and consequence variables to form an evaluation system of public satisfaction.

## 3. Material and Methods

### 3.1. Research Variables

This paper proposes 10 research variables and 25 measurable variables based on the ACSI model.

#### 3.1.1. Public Demand

This variable is derived from customer expectations in the ACSI but with some modification. Customer expectation refers to the customer’s expectation before purchasing of the level of quality when using the product or enjoying the service. However, since COVID-19 is a new pandemic, the public could not have had quality expectations, and only after they have experienced community services do they develop expectations for subsequent services [23]. Therefore, this variable was changed to public demand, referring to the public’s expectations and needs for the community in the subsequent COVID-19 services. Referring to ACSI’s customer expectation variable, the measurable variables are proposed, as shown in Table 1.

#### 3.1.2. Quality Perception

This variable is derived from the perceived quality of the ACSI model, which refers to the actual feelings of customers about the quality of a product or service after they have used it or enjoyed it. Quality perception is the public’s intuitive overall perception of the quality of community COVID-19 services after experiencing them. According to the literature review in Section 2.1, current research on community services for COVID-19 includes service resources, service content, service access channels, and public participation, so quality perception can be measured by these four aspects, as shown in Table 2.

#### 3.1.3. Service Maturity

This variable is derived from the perceived value in ACSI, which refers to how customers perceive the value of a product when they buy and use it. This can be reflected in the community services for COVID-19 as a perception of service maturity. The maturity of community COVID-19 services depends on the scope of coverage of the population and geographical span on the one hand, and the richness and perfection of the service measures to encompass the needs of different groups of people on the other [30]. Thus, the measurable variables F1 and F2 are proposed, as shown in Table 3.

#### 3.1.4. Public Satisfaction

This variable is derived from ACSI’s customer satisfaction variable, which is basically the same but differs only in the use of scenarios. Fornell explains public customer satisfaction as the public’s feedback on whether they are satisfied after enjoying a product or service based on their actual experience [21]. Referring to ACSI’s customer satisfaction variable, the G1 and G2 measurable variables are proposed, as shown in Table 4.

#### 3.1.5. Impact Results: Service Image and Public Trust

In the ACSI model, customer satisfaction has an impact on customer loyalty and customer complaints. Most communities in China have some degree of monopoly over community services. Most of the services provided by the community to its residents (e.g., infrastructure within the community) are unique, have no competitors, and are restricted by law and regulation to be “exclusive” so that people cannot obtain them elsewhere. If the public decides to accept the community’s services, the scope of access is limited to the framework of the community. Public loyalty and complaints about the community have been less studied for their value. According to Wu C, Shi Z, and Wilkes R [25], public satisfaction with the handling of the COVID-19 pandemic has an impact on service image and public trust, and this paper therefore proposes service image and public trust as variables affecting the results and establishes measurable variables, as shown in Table 5. Service image was explored in relation to the overall image [31], work efficiency [32], public monitoring [33], and public response [34] and public trust were explored in relation to the level of trust and support of the public in the community after experiencing COVID-19 services [27].

### 3.2. Model and Hypotheses

**Figure 2:**
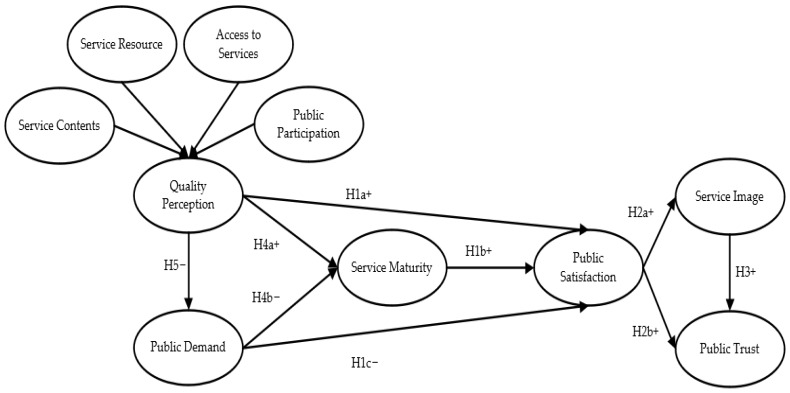
The public satisfaction model of community services for COVID-19.

According to the ACSI model, used to explore the relationship between the influence of each potential variable on satisfaction, a model of satisfaction with community services for COVID-19 was formed, as shown in Figure 2.

#### 3.2.1. Factors Influencing Public Satisfaction

According to the ACSI model, public satisfaction is influenced by perceived quality, perceived value, and customer expectations. The higher the residents’ perception of the quality of community COVID-19 services, the higher the satisfaction and also the higher the perceived value of the services, which in this paper means the higher the service maturity. Therefore, research hypotheses 1a and 1b are proposed. However, the greater the customer demand for service needs, the more likely it is that the satisfaction will decrease, so research hypothesis 1c is proposed.

**H1.**  *Public satisfaction is positively influenced by quality perception (a) and service maturity (b) and negatively influenced by public demand (c).*

#### 3.2.2. Effects of Public Satisfaction

The satisfaction of residents with the community services for COVID-19 affects their subsequent choice of and cooperation with the services. If residents are more satisfied with the service, they will continue to support community-related services and have more trust, and the image of the community in the eyes of the residents will be continually established and strengthened. Accordingly, hypotheses 2a and 2b are formulated. In addition, there is a relationship between service image and public trust. If the community has a better service image and leaves a good impression on the residents, the residents will trust it to be able to provide high-quality COVID-19 services, and their support for the work of COVID-19 services will be enhanced, according to which hypothesis H3 is proposed.

**H2.**  *Public satisfaction positively affects service image (a) and public trust (b).*

**H3.**  *Service image positively influences public trust.*

#### 3.2.3. Factors Influencing the Service Maturity

According to the ACSI model, perceived quality and customer expectations influence perceived value. In this study, the service maturity originates from the perceived value, which is influenced by quality perception and public demand, whereby the better the perceived service, the higher the maturity, but the greater the public demand, the lower the maturity. Therefore, research hypotheses 4a and 4b are proposed.

**H4.**  *The maturity of prevention and control services is positively influenced by quality perception (a) and negatively influenced by public demand (b).*

#### 3.2.4. Factors Influencing Public Demand

In the ACSI model, perceived quality has a significant negative effect on customer expectations. However, in this paper, public demand indicators are used, referring to the public’s further expectations for subsequent COVID-19 services, so it is considered that the perceived quality of the service affects subsequent demand, and the better the perceived quality, the weaker the demand should be. Therefore, research hypothesis 5 is proposed.

**H5.**  *Public demand is negatively influenced by public perception.*

### 3.3. Data Source and Analysis Method

#### 3.3.1. Case Selection

This paper selected a community in Beijing named “D” as a case study, located in the Southeast of Haidian District. In this community, there are 38 buildings, including 27 residential buildings, 183 units, 2548 households and a total of approximately 6500 people in the community of which approximately 2700 are a floating population. Residents are mainly represented by cadres, workers and their families, and retired cadres of the Air Force Logistics Department, Haidian Construction Committee, Academy of Post and Telecommunications, Six Institutes of the Ministry of Electronics, National Natural Fund Committee, Education Press, National Examination Center, China Meteorological Bureau, Tsinghua Tongfang company (THTF), Second Spinning Machinery Factory, etc. (data source: interview with the D community committee). This community was chosen for two reasons. First, Beijing, as the political and economic center of China [36], is at the forefront of community services for COVID-19 in the country’s cities, and because several confirmed cases of COVID-19 pandemic and close contacts were found in Beijing’s local areas in February 2021, Beijing has again increased its pandemic prevention and control efforts and strengthened prevention and control management in residential neighborhoods and other areas. Additionally, residents have a higher degree of memory when it comes to community services for COVID-19 [37]. Second, community “D” in Beijing has had more than one case of close contact with confirmed patients, so the community has strengthened its pandemic prevention and control management [38]. Furthermore, because of its proximity to Beijing universities and Zhongguancun Venture Park, most of its residents have a higher education level than ordinary communities, a higher acceptance of the survey questionnaire, and can reflect the current situation of community services for COVID-19 more objectively and honestly, which is typical of highly educated communities in Beijing and has guiding significance for similar urban communities with highly educated residents. Therefore, “D” community was selected as the main location in which to conduct the study.

#### 3.3.2. Distribution and Recovery of Questionnaires

This study was based on a sampling questionnaire survey. The research questions in the questionnaire were set according to the research variables in Table 1, Table 2, Table 3, Table 4 and Table 5, and answers to all questions were designed according to the Likert five-level scale (from 1 to 5, representing least conforming to most conforming). The following three steps were used to conduct the sampling survey:

1. Number of questionnaires

According to the requirements in terms of sampling proportion and the funding support of the paper, the paper set the sampling proportion at 7%, and decided to distribute 450 questionnaires;

2. Stratified sampling

Stratified sampling refers to dividing the overall sample into several groups according to certain characteristics (such as age, sex, etc.) that have a great impact on the observation indicators, and then randomly sampling each group. A certain number of observation units are randomly selected from each group, and the observation units selected at each level are combined to form a sample.

In this study, 450 questionnaires were distributed based on the age ratio of residents obtained from the interview with the “D” community committee. The number of questionnaires distributed at each age stage is shown in Table 6.

3. Sample collection

The questionnaires were distributed online through Questionnaire star (an online questionnaire website) over a period of approximately 2 months, from 10 May 2021 to 5 July 2021. The investigator joined the resident chat group of D community built by the community committee on Wechat (a mobile instant text and voice messaging communication application and an important social media platform in China [39]) to solicit respondents. The number of respondents collected for each age is in accordance with the number of distributed samples in Table 6. An online link on “Questionnaire Star” was sent to each respondent to fill in. “Questionnaire Star” can automatically collect questionnaires and the data for analysis also can be download from it.

A total of 423 questionnaires were eventually collected, with a response rate of 94%. The invalid questionnaires with incomplete and unclear answers, a single choice of options, or that were obviously unconventional or false were excluded, and the number of valid questionnaires totaled 372, with an effective response rate of 82.67%. The number of effective recovery questionnaires and the proportion of effective sample at each age group are shown in Table 6.

According to the statistics related to the questionnaire, community D is consistent with a highly educated urban community in which most (77.68%) of the residents are college-educated/have a bachelor’s degree or above in terms of demographic structure, and the obtained sample data strongly has the characteristics of a highly educated community, which has practical significance for the subsequent research analysis.

#### 3.3.3. Analysis Method

The structural equation model (SEM) is a statistical method used to analyze the relationship between variables based on their covariance matrix. It is an important tool for multivariate data analysis. If there is an interaction between independent variables, the regression model is not applicable, and SEM is more suitable for analysis. This study will use SEM to verify the model of public satisfaction with community services for COVID-19. Amos 22.0 software is used to analyze the structural equation of the model and verify the proposed hypotheses.

### 3.4. Reliability and Validity

Before the SEM analysis, the reliability and validity of the questionnaire data should be tested first. Cronbach’s alpha is usually used to test the reliability of a questionnaire. When the Cronbach’s alpha indicator of the scale is higher than 0.7, it indicates good agreement for multiple indicators of the concepts measured by the scale. The questionnaire data show that Cronbach’s alpha was 0.889, and Cronbach’s alpha coefficient values corresponding to the 10 dimensions of the scale were all greater than 0.7, indicating that the internal consistency of the questionnaire was good, so the reliability of the results of this survey was excellent. At the same time, the Cronbach’s alpha values for most of the items that had been removed from the entries were lower than the overall Cronbach’s alpha reliability coefficients for the corresponding dimensions; therefore, all questions were measures of the same concept and there was no need to remove items.

This study tested the correctness of the measurement scale by validity, using structural validity to test the intrinsic structure between the scale measures. The purpose of construct validity is to analyze the degree to which the 25 measures in the measurement scale explain the 10 factors in the model. The questionnaire data show that the KMO values of all measurable variables were greater than or equal to 0.5, which indicated the validity of the collected data, and the cumulative total variance explained of each measurable variable was greater than 60%, which indicated that the information content of the study items could be effectively extracted. In this study, CR values and AVE values were taken to evaluate the convergent validity. Convergent validity is generally considered good, when the CR value of each factor is greater than 0.7 and the AVE value is greater than 0.5. A high discriminant analysis validity is indicated when the square root of the AVE value of each factor is higher than the correlation coefficient between that factor and the other factors. The AVE values of all 10 dimensions were greater than 0.5, and the CR values were greater than 0.7, indicating a high convergent validity of this dimension. The square root of the AVE values was greater than that of the correlation coefficient values with the other factors, so the discriminant validity between the factors within each variable was superior, indicating that the questions in the questionnaire were differentiated and that the questionnaire itself was scientific and could ensure the validity of the subsequent study.

### 3.5. Model Fitting

On the basis of previous studies, the absolute fit indexes CMIN/DF, GFI, and RMSEA, and the relative fit indexes NFI, CFI, and TLI were selected as the fitting standards in this study. The questionnaire data was imported into Amos 22.0, and the maximum likelihood estimation method was used to analyze the questionnaire data, obtaining the fitting index of the model, as seen in Table 7.

According to Table 7, the chi-squared value of this model is χ2 = 420.344 (DF = 262), that is, CMIN/DF = 1.6045 < 3, indicating that this test model has basically been fitted with the actual measurement object. The upper 90% confidence interval of RMSEA is 0.04, below 0.05, indicating that the model fits well. NFI = 0.906, which meets the general common recommendation to accept the model at values above 0.9; CFI = 0.962 > 0.9, TLI = 0.957 > 0.9 and IFI = 0.963 > 0.9. The above model fitness indexes all meet the measurement-related criteria, so the model fitness is high and the fit is found to be good through validation.

## 4. Results

### 4.1. Model Validation

**Figure 3:**
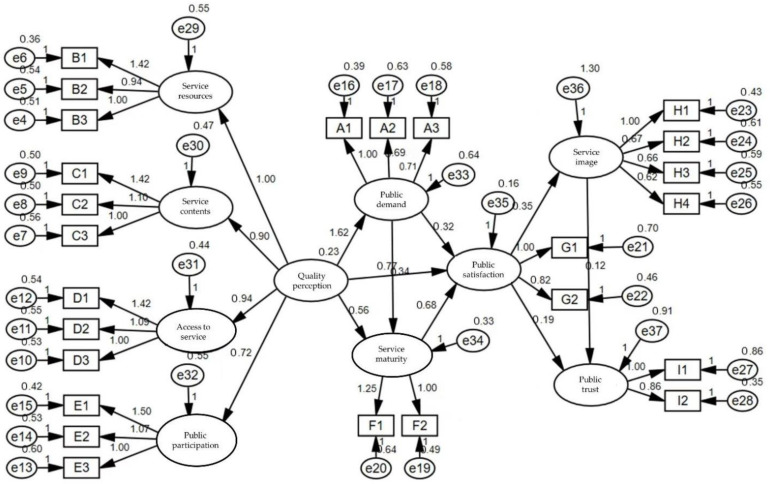
The public satisfaction model in a highly educated community.

The results of the path coefficient analysis of SEM in IBM SPSS Amos 22 (Source: IBM, New York, NY, USA) are shown in Figure 3.

### 4.2. Results Analysis

**Figure 4:**
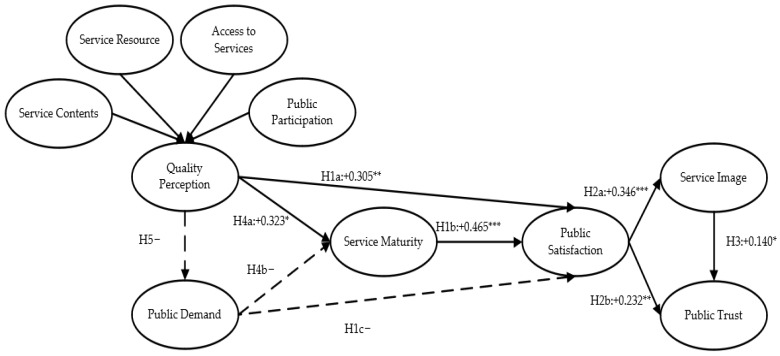
The results of the public satisfaction model. * *p* < 0.05; ** *p* < 0.01; *** *p* < 0.00.

A summary of the relationship paths among the variables is shown in Table 8. The results of this paper effectively validate part of the proposed research hypotheses, and the validation results of each research hypothesis are shown in Figure 4.

#### 4.2.1. Analysis of Factors Influencing Public Satisfaction

Public satisfaction with community services for COVID-19 is influenced by quality perception, public demand, and service maturity. The most significant impact on public satisfaction was found for the maturity of COVID-19 services, with a coefficient of 0.465, followed by quality perception, with a coefficient of 0.305. The least significant impact was found for public demand, with a coefficient of 0.295. The effects of these variables were all positive and all significant at the *p* < 0.05 level; therefore, hypotheses 1a and 1b hold, while hypothesis 1c does not, as shown in Figure 4. It is concluded that, first, improving quality perception can lead to increased satisfaction with community services for COVID-19. Second, all three variables have a certain degree of influence on public satisfaction with COVID-19 services in urban communities, and the degree of influence is in the following order: maturity of services, perceived quality, and public demand.

#### 4.2.2. Analysis of Effects of Public Satisfaction

Consequence factors of public satisfaction with COVID-19 pandemic services in highly educated urban communities are reflected in community service image and public trust. The coefficient of the effect of public satisfaction on community service image was 0.346, and the coefficient of the effect on public trust was 0.232. These effects were positive and both were significant at the *p* < 0.05 level, verifying that hypotheses 2a and 2b hold, as shown in Figure 4. This indicates that the higher the public satisfaction, the higher the service image of the community, and, in turn, the higher the public trust, where public satisfaction has a weak effect on public trust and a strong effect on service image. This also shows that improving public satisfaction can shape the overall image of the community as a provider of prevention services and increase the level of public trust.

Additionally, there is a relationship between the consequential factors of public satisfaction with the community services for the COVID-19 pandemic, with a significant positive effect of service image on public trust at the level of *p* < 0.1, with an effect coefficient of 0.140; research hypothesis 3 therefore holds. This shows that establishing a good community service image can effectively improve public trust and gain public support.

#### 4.2.3. Analysis of Factors Influencing Service Maturity

The maturity of community-based COVID-19 pandemic services was influenced by two variables. First, it was positively influenced by quality perception at the level of *p* < 0.1, with an influence coefficient of 0.323, which verifies the validity of hypothesis 4a, indicating that the higher the quality perception, the higher the service maturity. Second, it is positively influenced by public demand at the level of *p* < 0.01, with an impact coefficient of 0.460, so hypothesis 4b is not valid since the impact is positive and the hypothesis is negative.

#### 4.2.4. Analysis of Factors Influencing Public Demand

As shown in Table 8, perceived quality positively and significantly affects public demand at the level of *p* < 0.01, with an effect coefficient of 0.693; however, hypothesis 5 holds that perceived quality negatively affects public demand, so hypothesis 5 is not valid. This indicates that the higher the perceived quality, the higher the public demand.

## 5. Discussions and Limitations

### 5.1. Summary of Major Findings and Contributions

#### 5.1.1. Major Findings

There are two significant findings in this paper. The first is the validation of the antecedent variables of public satisfaction and the relationship among these variables in the scenario of community services for the COVID-19 pandemic. The results show that public satisfaction is significantly and positively influenced by quality perception (0.305 **), public demand (0.295 **) and service maturity (0.465 ***); service maturity is positively influenced by public demand (0.460 ***) and quality perception (0.323 *); and public demand is positively influenced by quality perception (0.693 ***). The second is to verify the consequence variables of public satisfaction and the relationship among these variables in the scenario of community services for the COVID-19 pandemic. The results show that public satisfaction has a significantly positive effect on service image (0.346 ***) and public trust (0.232 **), and service image significantly affects public trust (0.140 *).

#### 5.1.2. Contributions

On the one hand, this study enriches the research on community services for COVID-19. Current research on community services for COVID-19 focuses on four areas: community service resources [3,4,5,6], community service content [4,7,8], community service access channels [10], and how the public participate in community services [17,18,19]. This paper analyzes the public quality perception of community services for the COVID-19 pandemic in these four areas, and again validates the importance of these components. The theoretical contributions on this basis are three-fold: (1) the four aspects are studied in an integrated manner instead of focusing on only one aspect, which is found to constitute the community service system for the COVID-19 pandemic; (2) most researches are studied from the perspective of the community, and the variables of public satisfaction, public demand and public trust proposed in this paper are studied from the perspective of the public, which complements existing studies; and (3) this paper adds a case study in a highly educated community.

On the other hand, this study also enriches the research on public satisfaction. The ACSI model proposed by Fornell et al. in 1996 [21] suggested that the antecedent variables of merchandise customer satisfaction are perceived quality, customer expectations and perceived value, and the consequence variables are customer complaints and customer loyalty. Although some scholars have applied this model to the study of public satisfaction with government services [10,22], it has been less frequently applied to public satisfaction with community services. In this paper, the ACSI model is validated in the scenario of community services for COVID-19. The antecedent variables proposed include quality perception, service maturity (from perceived value) and public demand (from customer expectation), and the consequence variables proposed include public trust (from customer loyalty) and service image (from customer complaints). This paper makes the following three contributions to the application of the model in the scenario of community services for COVID-19: (1) The effect of public satisfaction on the consequence variables in the scenario of COVID-19 pandemic community service and the relationship among the consequence variables are consistent with the ACSI model. (2) Among the antecedent variables, only the positive effect of perceived quality and perceived value on public demand is verified, and the effect of public demand (from customer expectation) in the ACSI model on public satisfaction and service maturity (from perceived value) is shown to be negative, but this study found that this effect was positive in the highly educated community services for COVID-19. The reason for this may be that the public has a demand for service quality improvement, not because they are dissatisfied with the quality of services or think that the maturity level is not high but because they have their own suggestions for public services based on being highly educated community-dwelling people, who have their own knowledge and opinions about COVID-19 services due to their high level of knowledge. (3). In the relationship between the antecedent variables, the ACSI model suggests that customer expectations have a significant negative effect on perceived quality. Customer expectations are the expectations of service quality prior to using the service. Since the COVID-19 pandemic is novel and was not previously expected, this paper changes it to the indicator of public demand, which refers to further public demand for subsequent COVID-19 pandemic services. Customer expectation and public demand differ in the time of using the service. Customer expectation is determined before using the relevant service and public demand is after using the relevant service, so public demand should be influenced by the perceived quality, rather than influencing the perceived quality, as customer expectation does. Therefore, this study concluded the research hypothesis that perceived quality has a significant negative impact on the public’s subsequent demand. However, the results showed a positive impact. This may be because according to Maslow’s hierarchy of needs theory, people will pursue higher-level needs after satisfying lower-level needs, and this phenomenon will be more pronounced among residents of highly educated communities. Hence, the public, after experiencing and perceiving the quality of the existing COVID-19 pandemic services, has their basic needs met but will continue to increase their desires and needs and pursue higher level needs, expecting a higher level of service quality, such as more refined and unique services in the pandemic. Thus, public demand is positively influenced by perceived quality.

### 5.2. Limitations and Suggestions for Future Research

This study also has the following shortcomings: First, this study only selected one highly educated community (“D” community in Beijing) to conduct a survey of resident satisfaction, which may have been one-sided. However, this community, as a representative highly educated community in Beijing, can reflect the general characteristics of highly educated community residents to a certain extent. Second, due to constraints, such as pandemic control and funds, only 450 questionnaires based on the age ratio were issued. This may have resulted in an insufficient sample size and an inability to continuously observe changes in the demand for COVID-19 pandemic services in this community. This paper also proposes the following research prospects: first, in the follow-up study, the number of samples can be expanded by selecting samples from other highly educated communities; second, according to the situation in terms of pandemic prevention and control, questionnaires can be distributed both in stages and continuously to obtain more complete opinions of community residents during the COVID-19 pandemic.

## 6. Conclusions and Practical Suggestions

### 6.1. Understanding and Meeting Public Demand

According to the results of the paper, the public demand for community services for COVID-19 had a significant positive impact on both community service maturity and public satisfaction. This requires the community to understand and meet the needs of the public in a timely manner in pandemic services, which can be achieved in two ways.

On the one hand, the community should actively promote and guide residents toward a correct understanding of urban community services for COVID-19, so that residents’ psychological expectations are in line with their normal needs and do not create unattainable public demands; service providers should adhere to integrity, establish a good public image, not exaggerate their true capabilities and service levels, and not easily promise and imply that residents form unattainable expectations.

On the other hand, because public demand is significantly and positively influenced by the public’s perception of service quality, the content of COVID-19 services in urban communities should be continuously improved, by approaches, such as increasing the provision of pandemic prevention materials and ensuring the continuity of the supply of pandemic prevention materials; actively promoting the importance of pandemic prevention and related measures; and guiding residents to receive vaccinations. Highly educated urban communities should broaden residents’ access to COVID-19 services, strengthen digital urban governance through a combination of online and offline approaches, and provide convenient and efficient service access and feedback channels.

### 6.2. Enhancing the Public’s Quality Perception

According to the results of this paper, the public perception of community service quality significantly affects public satisfaction, service maturity and public demand, and plays a very important role in the model of public satisfaction with community services in the COVID-19 pandemic. According to the study, quality perception consists of four components—service content, service resources, service channels, and public participation—so it should be improved from the perspective of all four of these aspects.

Firstly, building and improving the content of community services for COVID-19 includes both pandemic prevention and control measures, such as daily community disinfection and sterilization and other medical and health services, the strict monitoring of people entering and leaving the community, and the isolation of close contacts, as well as complementary soft services, such as providing information and consultation management, pandemic prevention and control, vaccination promotion, calming isolated people, and strengthening mental health. Highly educated urban community residents have high demands in terms of mental health and a high mobility due to work, which can be empowered by modern communication and analysis technologies, such as Big Data, Internet of Things, and WeChat.

Secondly, to ensure that the community has sufficient supplies and to improve the relevant conditions and equipment, it is necessary to ensure the continuous provision of sufficient pandemic prevention and control materials, such as disinfectant, masks, etc., establish public infrastructure and crisis control places, and establish community-wide cultural and sports service centers, primary medical service centers, necessary isolation and control places, etc. At the same time, it is necessary to strengthen team building for community prevention and control, ensure sufficient personnel to provide prevention and control services, recruit community volunteers, conduct appropriate training, introduce social forces, and actively advocate cooperation between enterprises, institutions and communities for pandemic prevention.

Thirdly, in order to broaden and enrich the channels of pandemic prevention and control services in highly educated urban communities, attention must be paid to the personalized needs of different groups of people for channel access, through approaches, such as posting notices in places frequented by the elderly, empowering urban community prevention and control services with digital methods for young people, building modern information technology methods, such as electronic cash registers and community prevention WeChat groups, changing the previous single-channel approach and enhancing the two-way interaction between the service subject and the object.

Fourthly, public participation must be strengthened. The people living in highly educated urban communities are relatively knowledgeable, have a more objective and rational understanding of the pandemic, and have a higher degree of cooperation, so they are more likely to engage in public participation. Through a combination of online and offline methods, the timely notification of relevant information can be delivered in order to ensure citizens’ right to know. It is important to understand the actual needs of citizens, improve the institutional mechanism for resident feedback, reply in a timely manner and improve feedback content, and form a feedback system consisting of “experience-feedback-reply and solution-evaluation”. It is also important to advocate for residents’ personal participation in order for the public to understand and cooperate with community prevention work, reducing the pressure on community workers in terms of pandemic prevention.

### 6.3. Improving the Maturity of Community Services for COVID-19

According to the results of this study, the maturity of COVID-19 pandemic services in the community has a significant impact on public satisfaction. Therefore, in order to improve community health services, the maturity of services also needs to be improved, which can be achieved in the following two ways.

On the one hand, we should gradually refine and improve the service measures provided by the community. Although most of the people living in highly educated urban communities are people with a high level of education, their length of residence, professional backgrounds and geographical origins are quite different, and their worldviews, values and outlooks on life are also variable, which will affect the level of public needs and perceptions. Therefore, communities need to improve the quality and level of services to meet the demands of different people according to their different backgrounds.

On the other hand, the scope of community prevention and control should be gradually broadened, not only to provide pandemic prevention and control services to residents in the district but also to provide appropriate pandemic prevention information and mental health guidance to residents who are isolated by the pandemic, so that no resident can be neglected in terms of pandemic prevention and control. At the same time, it is important to encourage cooperation with the surrounding communities, enterprises and other nearby units for pandemic prevention and control, so as to improve the efficiency of pandemic prevention and control work in urban communities.

### 6.4. Promoting the Establishment of the Public Satisfaction Measure System

According to the results, the public satisfaction model of community services for COVID-19 was validated in a highly educated community in Beijing, indicating that the model can effectively measure public satisfaction with community services during the COVID-19 pandemic. This measurement can be administered on a regular basis in the following three ways.

Firstly, it is necessary to establish a management body for evaluating public satisfaction with pandemic prevention and control services in urban communities. Relevant experts and scholars should be gathered, especially experts in infectious disease prevention and control, resident representatives should be elected, and an objective and truthful assessment body should be established to evaluate and test public satisfaction with pandemic prevention and control services in urban communities. Public satisfaction evaluation need not be formal but must form a long-term mechanism that is both normalized and professionalized.

Secondly, the diversity and representativeness of evaluation subjects should be increased, and attention paid to the difference in and optimization of evaluation indicators across different periods. In the evaluation of service quality, the recipients of public services should be the mandatory evaluation subjects and should draw the vast majority of focus. At the same time, by paying attention to the regional and cultural differences in communities, the specific functions of community construction, the needs and work development of community services, and the differences in individual evaluation subjects, the evaluation indexes can be constantly adjusted and updated.

Thirdly, a scientific measurement model of public satisfaction with community services for COVID-19 should be established. The public satisfaction measurement system of highly educated urban community services for COVID-19 covers variables, such as the service quality perception of community residents, community image, service maturity, and satisfaction with community service organizations, which can not only refine various assessment criteria, optimize urban community service tools, and improve service quality but also meet residents’ needs and gain their trust and support.

## Figures and Tables

**Table 1:** Connotation and measurable variables of public demand.

Connotation	Measurable Variables
Whether the public has new needs for community services for COVID-19 after experiencing the services provided by the community.	A1 I think the overall community services for COVID-19 need to be improved [28].
	A2 I think the reliability of community services for COVID-19 needs to be improved [29].
	A3 I think community services for COVID-19 need to be improved in terms of meeting individualized needs [19].

**Table 2:** Connotation and measurable variables of quality perception.

Connotation	Indicators	Measurable Variables
Public perception of the overall quality of community services for COVID-19.	Service resources	B1 I think there is an adequate workforce for community COVID-19 services [3].
		B2 I think the infrastructure of community COVID-19 services is well developed [4].
		B3 I think there is sufficient financial support for community COVID-19 services [6].
	Service content	C4 I think the community services for COVID-19 are rich in measures [7].
		C5 I think the measures of community services for COVID-19 are scientific [8].
		C6 I think the measures of community services for COVID-19 are effective [4].
	Access to services	D7 I have easy access to community services for COVID-19 [10].
		D8 The access to community services for COVID-19 works for me [10].
		D9 I have much access to services for COVID-19 in my community [9].
	Public participation	E10 I participate actively in the community services for COVID-19 [17].
		E11 I think the community provides personalized COVID-19 services [19].
		E12 I think there are enough opportunities provided by the community to involve the public in COVID-19 services [18].

**Table 3:** Connotation and measurable variables of service maturity.

Connotation	Measurable Variables
Scope size and perfection of community services for COVID-19.	F1 I think the measures of community services for COVID-19 are perfect [10].
	F2 I think the scope of community services for COVID-19 is wide enough [7].

**Table 4:** Connotation and measurable variables of public satisfaction.

Connotation	Measurable Variables
Recognition of the services enjoyed by the public after they have experienced COVID-19 community services.	G1 I am satisfied with the overall community services for COVID-19 [21].
	G2 I think community services for COVD-19 are meeting my expected needs [23].

**Table 5:** Connotation and measurable variables of service image and public trust.

Variables	Connotation	Measurable Variables
Service image	The overall image of the community built by the public as they receive the COVID-19 services provided by the community.	H1 I think the overall image of community services for COVID-19 is good [31].
		H2 I think the community services for COVID-19 are working efficiently [32].
		H3 I think the community services for COVID-19 are well positioned to receive public complaints and scrutiny [33].
		H4 I think the community has responded to complaints or suggestions in a timely manner [34].
Public trust	The level of trust and support shown by the public for the community after experiencing the COVID-19 services.	I1 I trust the work of the community services for COVID-19 [35].
		I2 I am willing to cooperate with the work of the community services for COVID-19 [27].

**Table 6:** Number of questionnaires distributed and collected.

Age	Proportion of Total Population	Distributed Samples	Effective Samples Recovered	Proportion of Effective Sample
<18	3%	14	11	2.96%
18–25	18%	81	67	18.01%
26–45	49%	221	182	48.92%
46–60	21%	94	78	20.97%
>60 year	9%	40	34	9.14%
Total	100%	450	372	100%

(Data source for the proportion of total population: interview with the “D” community committee.)

**Table 7:** Fitting index of the public satisfaction model.

Indicator Type	Fitting Index	Evaluation Criteria or Thresholds	Model Estimation
Absolute Fit Index	CMIN/DF	<3	1.6045
	GFI	>0.8	0.920
	RMSEA	<0.08	0.04
Relative Fit Index	NFI	>0.8	0.906
	CFI	>0.9	0.962
	TLI	>0.8	0.957

**Table 8:** The path test.

Independent Variable		Dependent Variable	Unstandardized Regression Coefficients	Standardized Regression Coefficients	Standard Errors	T-Value	*p*-Value	Hypothesis
Quality Perception	---->	Public Satisfaction	0.774	0.305	0.265	2.918	**	H1a
Service Maturity	---->	Public Satisfaction	0.677	0.465	0.119	5.668	***	H1b
Public Demand	---->	Public Satisfaction	0.321	0.295	0.092	3.495	***	H1c
Public Satisfaction	---->	Service Image	0.348	0.346	0.059	5.920	***	H2a
Public Satisfaction	---->	Public Trust	0.193	0.232	0.061	3.171	**	H2b
Service Image	---->	Public Trust	0.115	0.140	0.057	2.019	*	H3
Quality Perception	---->	Service Maturity	0.562	0.323	0.221	2.546	*	H4a
Public Demand	---->	Service Maturity	0.344	0.460	0.081	4.265	***	H4b
Quality Perception	---->	Public Demand	1.616	0.693	0.262	6.174	***	H5

* *p* < 0.05; ** *p* < 0.01; *** *p* < 0.00.

## Data Availability

The dataset generated and analyzed in this study is not publicly available; however, the dataset is available from the corresponding author upon reasonable request.
